# From scar to stone: Enterolith formation in an ileal stricture post-appendectomy – A case report and literature review

**DOI:** 10.1016/j.ijscr.2025.111694

**Published:** 2025-07-16

**Authors:** Nikhil Agrawal, Bishal Khadka, Devendra Bist

**Affiliations:** a Kathmandu Medical College and Teaching Hospital, Sinamangal, Kathmandu, Nepal; b Department of General Surgery, Kathmandu Medical College and Teaching Hospital, Sinamangal, Kathmandu, Nepal

**Keywords:** Abdominal pain, Calculi, Case report, Enterectomy, Intestinal obstruction, Stricture

## Abstract

**Introduction:**

The enteroliths are stones of the intestines that may form along the passage of the gastrointestinal tract, especially within the ileum. Their clinical and radiological presentations vary, often leading to misdiagnosis. They may result in delayed complications like intestinal obstruction.

**Case presentation:**

We present a case of enterolithiasis secondary to ileal stricture post-appendectomy, in a 69-year-old male, which was then treated successfully via segmental ileal resection with enterolith extraction and ileoileal anastomosis in our tertiary center after thorough examination.

**Discussion:**

This case highlights how a seemingly routine post-surgical history can hide a rare condition like enterolithiasis. In our patient, chronic abdominal discomfort after an old appendectomy turned out to be due to a stone formed in the small intestine because of a stricture. It reminds clinicians to consider atypical etiologies when symptoms persist, and to review past surgeries as potential contributors to delayed complications.

**Conclusion:**

Enteroliths are caused by altered bowel state and may seldom cause bowel obstruction. Imaging usually suffices for diagnosis but mimics must be ruled out. Treatment includes endoscopic or surgical removal of enterolith and addressing any causative factors.

## Highlights

• This case underscores the rare occurrence of primary enterolithiasis, secondary to a post-surgical ileal stricture, two decades after an appendectomy.
• Radiological clues were critical to raise suspicion for enterolithiasis.
• Surgical management ensures complete resolution.
• Literature review supports that stasis from strictures- whether inflammatory, neoplastic, or post-surgical-remains a key driver in enterolith formation.

## 1 Introduction

Enterolithiasis is a significant but relatively rare clinical condition that has received renewed clinical interest with advances in the gastrointestinal field [1,2]. The prevalence of this condition lies between 0.3 % and 10 % though true rates may be higher due to underdiagnosis [3,4]. Enteroliths are classified into primary and secondary based on their formation [5,6]. The mortality rate in uncomplicated primary enterolithiasis is extremely low, however in severe conditions in patients with significant obstruction and late diagnosis, it goes up to 3 % [[7], [8], [9]].

The mortality of secondary enteroliths reach up to 8 % [10]. Presentation is often nonspecific, but typically includes “tumbling” abdominal pain, nausea, and vomiting related to the bowel obstruction, and may potentially lead to gastrointestinal bleeding and perforation [5,8]. The case report has been reported in line with the *SCARE* criteria [11].

## 2 Case presentation

A 69 years old male, post-appendectomy status (22 years back), presented to the surgery department with a history of intermittent pain over the right iliac fossa since surgery, recently worsening in intensity. The pain was dull, non-radiating, relieved by medications with no associated symptoms. He visited multiple centers over the years, was managed with medication for pain and was dismissed. He was admitted for further evaluation and treatment. He does not have any comorbidities, motility disorders and family history was insignificant for similar illnesses. He consumes a typical mixed Nepalese diet. There is no history of foreign body ingestion.

On admission, he was afebrile but tachycardic (110 bpm) and hypotensive (60/40 mmHg), with respiratory rate of 24 breaths per minute. Abdominal examination revealed a well healed right lower quadrant scar, 8 cm in length consistent with open appendectomy, mild generalised tenderness and sluggish bowel sounds.

Laboratory tests on Day 1 of admission indicated moderate anemia (Hb: 9.5 g/dL) likely due to chronic inflammation, neutrophilia (79 %), and elevated blood urea (39 mg/dL). The laboratory investigations were otherwise unremarkable. The patient received IV fluids and electrolytes for dehydration. Elevated urea was likely secondary to pre-renal azotemia from dehydration. Serum creatinine was within normal limits (1.1 mg/dL).

**Fig. 1:**
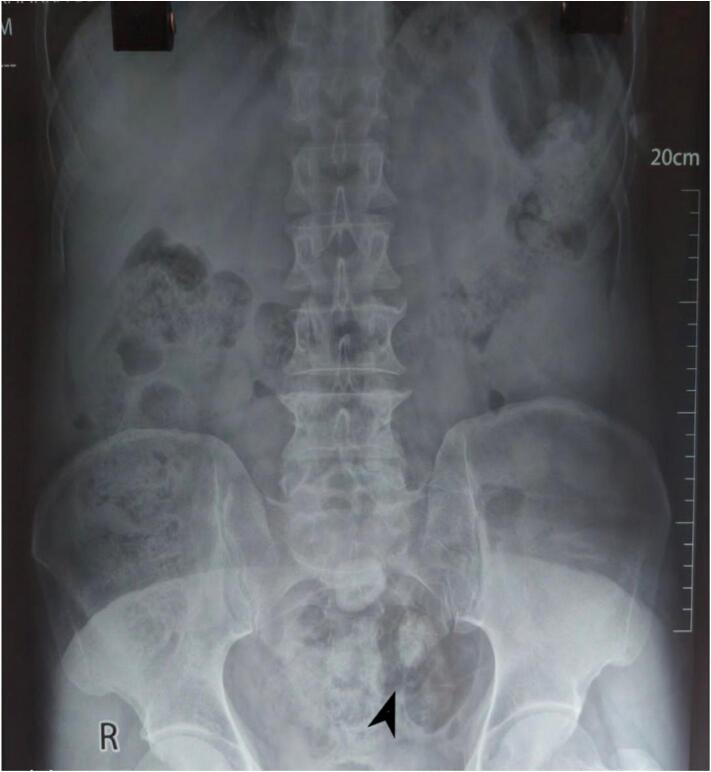
Erect plain abdominal radiograph showing a round radiopaque shadow (black arrow) suggestive of enterolith.

**Fig. 2:**
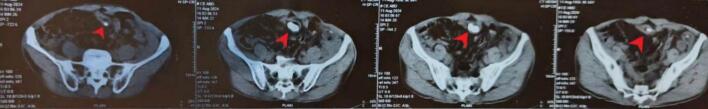
Contrast-enhanced computed tomography (CECT) of the abdomen showing a ring-like hyperdense lesion in the terminal ileum (red arrows) consistent with a radio-dense enterolith. (For interpretation of the references to colour in this figure legend, the reader is referred to the web version of this article.)

X-ray revealed a well-defined round radiopacity localized in the suprapubic region (Fig. 1). Colonoscopy visualized till hepatic flexure with no specific findings. On Contrast Enhanced Computed Tomography (CECT) (Abdomen plus Pelvis), an abnormal ring-like hyper-density in the terminal bowel lumen was observed (Fig. 2). The ring-like hyper-density was suspected to be an enterolith that was causing the pain. Mild bowel obstruction was anticipated.

After detailed evaluation, the patient was admitted and planned for an exploratory laparotomy for removal of suspected enterolith, the next day. Under spinal anesthesia, a midline incision was given after all the aseptic and preoperative procedures. Adhesiolysis was done for a better visual field. Proximal bowel was mildly dilated and the distal bowel mildly collapsed. Dense adhesions were present between the bowel and the anterior abdominal wall.

**Fig. 3:**
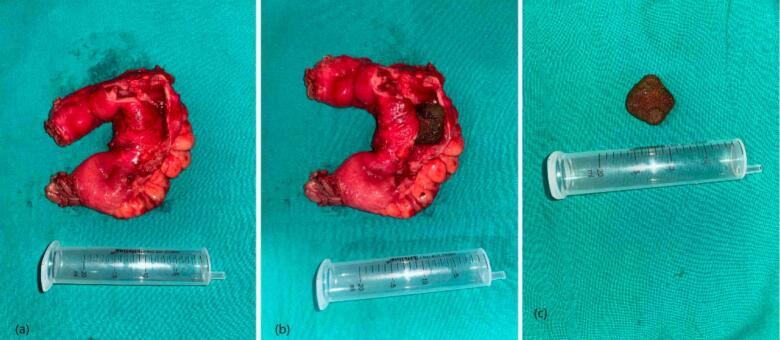
(a) Resected segment of ileum, (b) Intraoperative image showing the enterolith within the ileum and (c) Removed enterolith measuring 2*1 cm.

Enterolith measuring approximately 2 × 1 cm size was found (Fig. 3c), 160 cm distal to the duodeno-jejunal flexure (DJ flexure) where stricture was also present. Dense adhesions and localized fibrotic changes were seen near the strictured segment, indicating chronic post-surgical inflammation rather than an intrinsic bowel pathology. The enterolith with the stricture was removed (Fig. 3), and ileoileal anastomosis, using Barcelona functional technique with GastroIntestinal Anastomosis (GIA) staplers. The enterolith and the resected segment were not sent for analyses due to lack of facilities and patient's economical reasons. A drain was placed in the pelvis and the patient was shifted to the high care unit. Patient was kept Nil Per Oral (NPO) for 6 h, then a clear liquid diet was started. His postoperative period was uneventful. Abdominal drain was removed on the 6th Post-Operative Day (POD). He was then discharged with some medications after counseling. Follow-up continued for 3 months post-discharge without recurrence.

## 3 Discussion

Enteroliths are intestinal concretions that may be asymptomatic or cause inflammation and obstruction [4,6,12,13]. Enteroliths can vary in size and quantity, ranging from a few millimeters to several centimeters and can be solitary or multiple [1,8]. Enteroliths are classified into two types: primary (true) and secondary (false). Primary enteroliths develop in the small or large intestine. It is often linked to underlying conditions that cause reduced movement and stasis in the intestines [5]. Their composition can vary depending on the location as they are formed from the chemical components present in the bowel at compromised areas of stasis [14]. Diverticula, strictures, and low pH promote enterolith formation. Cholic acid stones form proximally in acidic pH, while calcium-based stones occur distally as they are soluble in water and acidic environments [15]. Likewise, in our case, the enterolith might have formed due to stasis caused by the stricture present approximately 160 cm distal to the DJ flexure, in the terminal ileum. We postulate that the stricture may have resulted from chronic adhesions due to a previous appendectomy. Although anatomically distant from the ileo-cecal valve, chronic post-appendectomy adhesions and localized inflammation may have resulted in stasis that likely created a favorable environment for enterolith formation [5,14].

Secondary enteroliths form outside the gastrointestinal tract and migrate to the bowel leading to its obstruction which maybe through a fistulous connection, as in gallstone ileus, where cholecysto-duodenal or cholecysto-jejunal fistula act as a path for the stone passage [9,16]. False enteroliths comprise of fecaliths, bezoars of any origin, and rarer entities like varnish or oat stones [17].

Strictures cause stasis which can be the primary cause in enterolith formation [7,18]. Stricture formation is associated with conditions like intestinal TB, carcinoid tumors, Crohn's Disease, post traumatic or post-surgical strictures, diverticula, radiation enteritis, etc [3,5,18]. This leads to favorable conditions for the accumulation and precipitation of ingredients of enteroliths developing in the small intestines [18]. In contrast, our patient had no history of TB, IBD or radiation exposure but had a history of previous appendectomy 22 years back.

Clinical presentation often varies and depends mostly on the etiology and location among other factors like age of the patient, dimensions and chemical composition of the stone [5,7,12]. True enteroliths are more likely in young individuals with tuberculosis or Inflammatory Bowel Disease (IBD), or elderly patients with a history of prior abdominal surgery, as seen in our case [1,3,4]. Small enteroliths are almost always asymptomatic and pass on their own [5,7]. Until and unless the symptoms direct towards the gastrointestinal tract, enteroliths may be mistaken for renal, ureteral or bladder stones according to their various locations. Bowel obstructions can be extramural (e.g., adhesions, hernia), mural (e.g., inflammatory conditions, strictures, tumors), or intraluminal (e.g., foreign bodies, parasites, bezoars). Intestinal colic due to enteroliths can sometimes be misinterpreted as symptomatic calculi in the urinary tract clinically as well as radiologically as seen here [19,20].

Detection of enterolith on plain abdominal X-ray depends on calcium content which enhances the visibility of the enterolith by increasing radio-opacity [9,19]. Distally located enteroliths have more content of calcium salts, as seen in our case [5]. Enterolith on plain abdominal X-ray usually shows a “coin on end” appearance, also observed in our case [19,20]. CECT usually reveals a void indicating a radiolucent stone. It significantly increases the yield of detection of radiolucent stones and may also help in identifying the number of enteroliths, their exact location and narrow the focus on the culprit stone [20]. Attention to the gallbladder and the biliary system aids to rule out gallstone ileus [5].

Optimal treatment of enterolithiasis must focus on enterolith removal followed by correction of underlying pathology which aids in the prevention of future formation of additional enteroliths [5,16]. In absence of underlying luminal compromise for stones less than 2 cm, expectant management with serial abdominal examinations, electrolyte correction and hydration along with nasogastric decompression may be considered in some cases [5,20]. Endoscopic segmental dilatation and stone retrieval may be reviewed first in cases of strictures, stenosis or anastomotic defects [16]. In various settings of Meckel's diverticulum, complicated strictures, significant inflammation, necrosis and perforations, segmental small bowel resection with intended primary anastomosis should be attempted [5]. Similarly, our patient with a stricture at terminal ileum was also managed through an exploratory laparotomy that involved removal of the enterolith with segmental ileal resection and anastomosis for the stricture.

This case underscores the importance of considering rare causes like enterolithiasis in patients with persistent abdominal symptoms post-surgery. Compared to previous cases in the literature, our patient lacked typical predisposing conditions like diverticula, Meckel's diverticulum, or active IBD, highlighting the diagnostic challenge posed by such unusual presentations [7,8].

## 4 Conclusion

Alterations in bowel anatomy and microenvironment plays a significant role in pathogenesis of this disease. These stones can be easily picked up by the CT scan of the abdomen, including the radiolucent ones which aids in the diagnosis of enteroliths. Enterolithiasis mimics should be carefully eliminated and treatment focus should be removal of the enterolith and any underlying pathologies.

## Author contribution

All authors contributed to the conception, writing and editing of the case report. All authors are agreed to be accountable for all aspects of it.

## Consent

Written informed consent was obtained from the patient for publication of this case report and accompanying images. A copy of the written consent is available for review by the Editor-in-Chief of this journal on request.

## Ethical approval

Not applicable.

## Guarantor

Devendra Bist.

## Research registration number

Not applicable.

## Sources of funding

This research did not receive any specific grant from funding agencies in the public, commercial, or not-for-profit sectors.

## Declaration of competing interest

None.
